# Idiopathic Pneumonia Syndrome and Thrombotic Microangiopathy Following Nonmyeloablative Haploidentical Peripheral Blood Stem Cell Transplantation and Posttransplant Cyclophosphamide: A Case Report

**DOI:** 10.1097/MD.0000000000001200

**Published:** 2015-07-24

**Authors:** Wei-Hsin Liu, Wei-Ting Chen, Li-Hua Fang, Rong-Long Chen

**Affiliations:** From the Department of Hematology and Oncology (W-HL); Department of Internal Medicine (W-TC); Department of Pharmacy (L-HF); and Department of Pediatric Hematology and Oncology (R-LC), Koo Foundation Sun Yat-Sen Cancer Center, Taipei, Taiwan.

## Abstract

Posttransplant high-dose cyclophosphamide (pT-HDCy) following T-cell-replete haploidentical bone marrow (BM) transplantation has been successfully utilized to control alloreactivity, mainly in ameliorating graft-versus-host disease (GVHD) and graft rejection. Recently, peripheral blood stem cells (PBSCs) have also been suggested to be a feasible and effective graft alternative to BM in the same setting.

We report a case with refractory Hodgkin lymphoma treated with haploidentical PBSC transplantation with nonmyeloablative conditioning and pT-HDCy. Although engraftment with complete donor chimerism was achieved without classical GVHD, the patient suffered from idiopathic pneumonia syndrome followed by thrombotic microangiopathy.

Although idiopathic pneumonia syndrome and thrombotic microangiopathy improved after treatment, the patient's lymphoma rapidly progressed nonetheless.

This outcome may suggest that the alloreactivity against the classical GVHD targets is successfully eradicated by pT-HDCy, but alloreactivity against the lungs and endothelial cells is differentially preserved when utilizing granulocyte colony-stimulating factor-mobilized PBSCs as the graft source. The graft-versus-Hodgkin lymphoma effect was not observed in our patient.

## INTRODUCTION

According to the pioneering work of Johns Hopkins University, haploidentical bone marrow (BM) transplantation with nonmyeloablative conditioning and posttransplant high-dose cyclophosphamide (pT-HDCy) has been successfully applied in the treatment of hematologic malignancies.^1^ BM is a preferred graft source in this setting because of the theoretical concerns that the larger number of T cells, typically present in granulocyte colony-stimulating factor-mobilized peripheral blood stem cells (PBSCs), may overwhelm pT-HDCy to suppress detrimental alloreactivity posttransplant. A recent report demonstrated that PBSCs can be safely and effectively utilized in haploidentical transplantations with similar nonmyeloablative conditioning and pT-HDCy to deliver results comparable to that of the BM transplantation in terms of engraftment, graft-versus-host disease (GVHD), and infection control.^2^ In addition, both BM and PBSCs have been demonstrated to be particularly effective as graft sources in the patients with poor prognosis Hodgkin lymphoma.^3–5^ Therefore, we applied the haploidentical PBSC transplantation strategy to treat a patient with Hodgkin lymphoma who had had relapses after 4 lines of treatments including autologous PBSC transplantation. She unexpectedly suffered from idiopathic pneumonia syndrome (IPS) followed by thrombotic microangiopathy (TMA) possibly related to nonsuppressed donor PBSC alloreactivity following pT-HDCy. Although she attained complete donor chimerism in the absence of classical acute GVHD, as seen in some reports, her tumor still rapidly progressed.

## CASE REPORT

**FIGURE 1:**
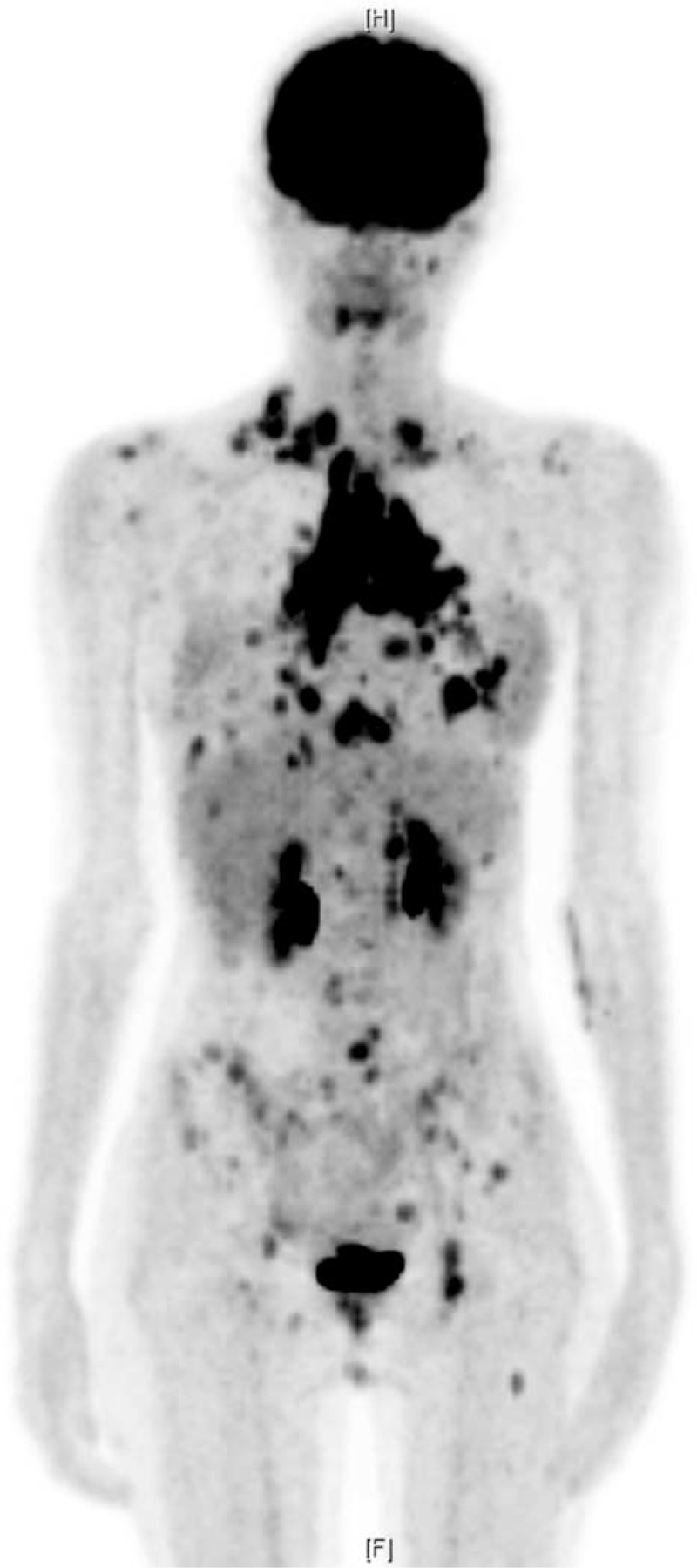
A positron emission tomography-computed tomography scan on July 18, 2014, revealed extensive [^18^F]fluorodeoxyglucose uptake within lymph nodes in the bilateral supraclavicular, mediastinal, pulmonary hilar, retrocardiac, cardiophrenic, gastrohepatic, abdominal paraaortic, left lower pelvic regions; bones in the left mandible, skull, spine, pelvic bones, bilateral scapulae, ribs, left femur; bilateral lungs and pleura; and pancreatic tail.

A 17-year-old girl was diagnosed with an initial stage IIB nodular sclerosis-type Hodgkin lymphoma in November 2012 at an outside hospital. She suffered from recurrent disease after 4 lines of treatments including autologous PBSC transplantation on December 8, 2013, after BCNU/etoposide/ara-C/melphalan conditioning. Intermittent coughs and epigastric pain was noted; she was transferred to our hospital where a positron emission tomography-computed tomography (CT) scan on July 18, 2014, revealed extensive disease (Figure 1). She subsequently received 3 cycles of gemcitabine/vinorelbine^6^ chemotherapy from July to September, during which she remained dyspneic from persistent thoracic lesions.

A decision was made for haploidentical PBSC transplantation with her elder brother as the donor. The conditioning treatment consisted of fludarabine (30 mg/m^2^ daily intravenous from day −6 to −2) and cyclophosphamide (14.5 mg/kg daily intravenous from day −6 to −5) as well as 2 Gy total body irradiation on day −1. For GVHD prophylaxis, the patient received pT-HDCy (50 mg/kg daily intravenous from day +3 to +4), whereas cyclosporine and mycophenolate mofetil were given from day +5. On October 7, 2014, she received PBSCs containing 14.5 × 10^8^ total nucleated cells/kg, 7.4 × 10^6^ CD34^+^ cells/kg, and 1.4 × 10^8^ CD3^+^ cells/kg. The patient and donor were human leukocyte antigen-5/10 matched (A 3101/2402, B 4001/1502, Cw 0304/0801, DQ 0301/0301, DR 1101/1202 to A 3101/0207, B 4001/4601, Cw 0304/0102, DQ 0301/0502, DR 1101/1454) and ABO nonidentical (B to A).

**FIGURE 2:**
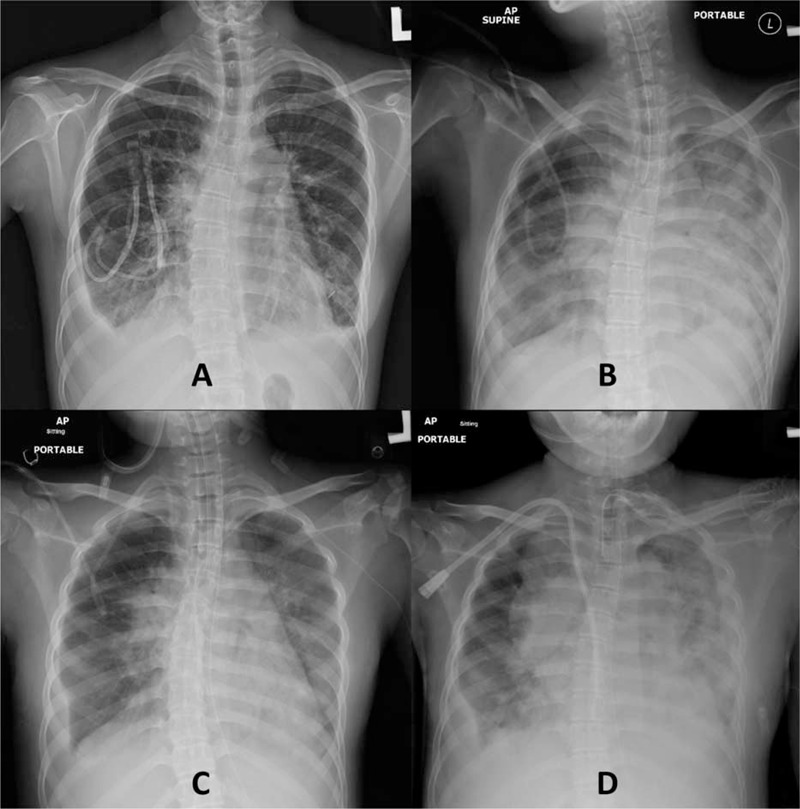
The serial chest x-rays perihaploidentical transplantation showing the following: (A) the appearance of bilateral pleural and pericardial effusions just after conditioning (day −1); (B) the rapid development of bilateral diffuse pulmonary infiltrates after engraftment (day +44); (C) the resolution of the pulmonary infiltrates after treatment with methylprednisolone and etanercept (day +50); (D) rapid tumor progression, including a mass in the neck, supraclavicular, axilla, mediastinum, lungs and pleura (day +83).

The postinfusion course was complicated by the rapid accumulation of pericardial and pleural effusions (Figure 2A) necessitating intermittent drainage. The fluid was serosanguinous without evidence of any microbial pathogens, whereas CD30/CD15/PAX-5^+^ cells were only detectable in the first of 2 pericardial (day +27) and last of 4 pleural (day +59) samples. Neutrophil engraftment with complete donor chimerism was documented on day +19. She received frequent red cell transfusions. She also received platelet transfusions every 2 to 3 days until day +32; thereafter, she did not require platelet transfusion until day +46.

However, cytomegalovirus reactivation was detected beginning on day +34. At the same time, reactivation of polyomavirus BK viruria caused grade III hemorrhagic cystitis. Epstein–Barr virus DNAemia was never detected throughout the entire course. She received ganciclovir (from day +36) along with intravenous immunoglobulins (a total of 7 doses from day +37 to day +69). Cytomegalovirus DNAemia was undetectable after day +55. However, the respiratory distress progressed and elective intubation with mechanical ventilation was started on day +44 (Figure 2B). IPS was diagnosed by negative microbial and cytology panel studies from repeated bronchoalveolar lavages on days +44 and +56, respectively, according to the definition updated by the American Thoracic Society Committee.^7^ The pneumonia improved after treatment with methylprednisolone along with etanercept (a total of 7 doses from days +44 to +69) (Figure 2C). No evidence of other microbial infections was documented throughout the entire course.

**FIGURE 3:**
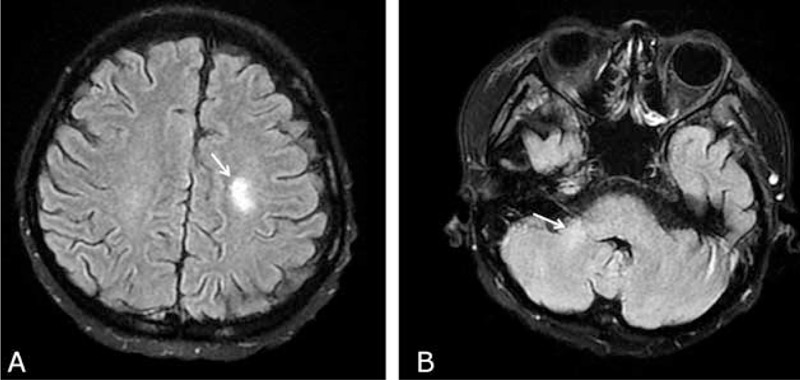
Brain magnetic resonance imaging on day +72 posttransplant showing T2-hyperintense lesions (arrows) in the (A) deeper site of left frontal lobe and in (B) right middle cerebellar peduncle.

After she was extubated on day +57, she still suffered from intermittent exacerbation of respiratory distress. Hypertension developed (from a baseline of 100/60 mm Hg until day +55 to >150/100 mm Hg after day +57) and renal function deteriorated (baseline blood urea nitrogen/creatinine of 56/1.05 mg/dL on day +54 elevated to 152/2.54 mg/dL on day +59). In addition, episodes of complex partial seizure were also noted. Multiple infarct-like lesions in the left frontal lobe, right middle cerebellar peduncle, and bilateral occipital white matter were found on day +72 by brain magnetic resonance imaging (Figure 3). TMA was diagnosed according to the proposed criteria^8^: marked elevation of lactate dehydrogenase (from 617 to 1074 U/L), aggravated anemia and thrombocytopenia, presence of schistocytes in the peripheral blood, absence of a coagulopathy, and a negative Coombs test. Tracheostomy was performed because of the unstable respiratory status on day +70. Cyclosporine A was discontinued from day +69. After supportive treatments, hemodialysis was discontinued after day +76 and no attacks of seizure/severe hypertension occurred after day +80.

Last, rapidly progressive hypercalcemia (from 9.4 mg/dL on day +73 to 13.7 mg/dL on day +82) was noted, and disease progression was documented by images including a CT scan on day +83, indicating abnormal lymph nodes at the bilateral lower neck, axilla, mediastinum, and retroperitoneum, and lesions at the pericardium, bilateral lungs, and hepatic lobes with progression of effusions (Figure 2D and data not shown). The patient and her family decided on hospice care and she finally passed away on day +94. Throughout the entire course, there was no evidence of classical acute GVHD whereas complete donor chimerism was documented until day +89.

## DISCUSSION

Our case report is consistent with the theory, as described by Bashey and Solomon,^9^ that application of pT-HDCy at the correct time following infusion of haploidentical donor cells can eliminate highly activated and proliferative T cells responsible for classical GVHD and graft rejection, whereas it spares the pluripotent hematopoietic stem cells in the graft. However, IPS and TMA still developed in our patient. Although cytomegalovirus infection of the respiratory as well as microvascular system cannot be fully excluded, the development of full-blown IPS and TMA despite the effective control of cytomegalovirus DNAemia suggests a major role for immune reactivity.

A mouse model using a major histocompatibility complex class I/II mismatched donor/recipient strain combination reproduces acute, early-onset IPS caused by the influx of host monocytes and donor alloreactive T cells into the lungs within the first 2 weeks of hematopoietic stem cell transplantation (HSCT). Intensifying the pre-HSCT conditioning with cyclophosphamide accelerates the development of IPS.^10^ This finding is consistent with the pulmonary microenvironment in our patient who had extensive pulmonary lesions and received irradiation along with cyclophosphamide-containing chemotherapy. Although the lung has not been traditionally recognized as a classical GVHD target organ, the association between IPS and severe GVHD has been reported with acute GVHD often preceding IPS.^11^ Therefore, the specific role of alloreactive donor T lymphocytes in the pathogenesis of IPS remains a debatable topic that has been extensively discussed in a published official research statement of the American Thoracic Society.^7^

Multiple factors contributed to the endothelial damage in TMA, including the toxic effect of the conditioning and cyclosporine; however, the alloreactivity from the T cells of the haploidentical PBSC graft must be of importance. Although the exact pathophysiology of transplantation-associated TMA remains unclear, it has been speculated that TMA represents a form of “endothelial GVHD.”^12^ Actually, acute GVHD has also been found to be associated with the development of TMA, indicating that donor alloreactivity may play an important role in transplantation-associated TMA.^13,14^

It seems that pT-HDCy successfully eliminated alloreactivity targeting the epithelial tissues of the skin, liver, and gastrointestinal systems, as classical acute GVHD was completely abrogated in our patient. The immunity to infections also seemed to be preserved because no other microbial infections were documented throughout the entire course except for cytomegalovirus and polyoma BK virus reactivation. However, the alloreactivity from the haploidentical donor PBSCs against the lungs and endothelial cells was not suppressed, which resulted in the development of IPS and TMA in our patient. This outcome may mean that when PBSCs are used for the graft in the context of nonmyeloablative conditioning, pT-HDCy may not be sufficient to eradicate detrimental alloreactive immunity targeting nonconventional acute GVHD sites such as the lungs and endothelial cells, despite the previously documented benefits of this method. Furthermore, the higher T-cell dose in donor PBSCs did not result in an adequate graft-versus-Hodgkin lymphoma effect in our patient.
